# Bladder Endometriosis Masquerading as Bladder Tumor: The Role of Magnetic Resonance Imaging in Diagnosis

**DOI:** 10.7759/cureus.16133

**Published:** 2021-07-03

**Authors:** Afshan Hakeem, Shayan S Anwar, Shahla S Anwar, Farwa Fatima, Anwar Ahmed

**Affiliations:** 1 Department of Radiology, Aga Khan University Hospital, Karachi, PAK; 2 Department of Family Medicine, Alberta Health Services, Red Deer, CAN

**Keywords:** bladder endometriosis, mri, t1 fat saturated sequence, t2 weighted sequence, ultrasound

## Abstract

Bladder endometriosis is an infrequent cause of a focal bladder mass and may masquerade as a neoplasm on ultrasound imaging. This diagnostic dilemma can be resolved with a multiparametric MRI, which shows characteristic hemorrhagic signals of this entity. We present an unusual case of bladder endometriosis where the patient complained of lower abdominal pain without urinary symptoms and was found to have a bladder mass on ultrasonography. This mass was further investigated by an MRI of the pelvis, which revealed characteristic features of endometriosis; the diagnosis was confirmed on subsequent laparoscopy.

## Introduction

Endometriosis is a commonly encountered chronic benign gynecological condition characterized by the presence of functional endometrial glands and stroma-like lesions outside the uterus. In rare cases, the involvement of the urinary system due to endometriosis has been observed. However, the most common site of the urinary system involvement due to this entity is the posterior wall of the urinary bladder, which is in close proximity with the uterus, and hence vulnerable to direct spread from the uterus [1]. In this report, we present a rare case of bladder endometriosis that mimicked a bladder tumor.

## Case presentation

A 31-year-old unmarried female presented with complaints of lower abdominal pain and severe dysmenorrhea. Her pain had gradually increased in intensity over time. Clinically, the patient was vitally stable, and her abdomen was soft and non-tender. A review of her records showed that she had undergone two previous ultrasounds; one had been performed nine years back in 2012, which had shown large multiloculated cystic lesions in both adnexa and cul-de-sac with low-level internal echoes suggestive of bilateral ovarian endometriomas. At least two intramural fibroids had also been identified in the uterine body. Subsequently, she had undergone a laparoscopic cystectomy, confirming endometrioma. She had later undergone laparotomy for myomectomy, adhesiolysis, and bilateral ovarian cystectomy. Her follow-up/second ultrasound had been done in 2016, which had shown new development of multiple uterine fibroids without any ovarian pathology.

**Figure 1:**
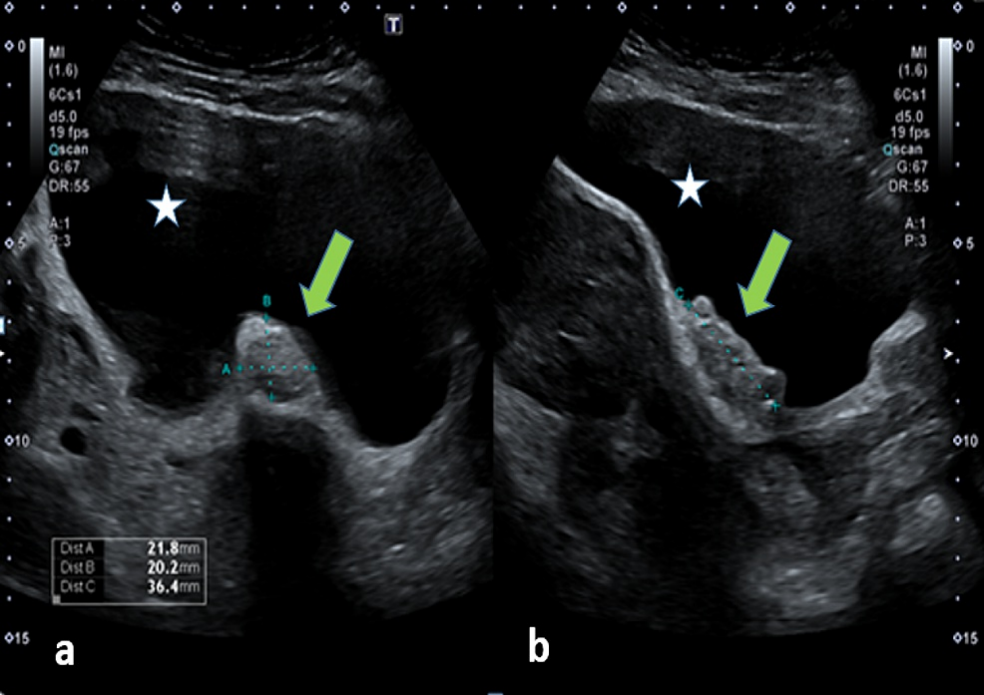
Ultrasound image (a) Transverse and (b) longitudinal image: a well-defined lobulated hypoechoic lesion (green arrow) along the base of the urinary bladder (star sign)

Based on her current symptoms, she again underwent an ultrasound pelvis after an interval period of five years, which demonstrated a bulky anteverted and anteflexed enlarged uterus with multiple intramural and subserosal fibroids of variable sizes. The urinary bladder base revealed a slightly lobulated well-defined hypoechoic immobile mass without any significant vascularity, measuring 22 x 20 x 36 mm (Figure 1). Both ovaries were normal, and no free fluid was seen in the pelvis. Based on ultrasound findings, the patient was advised cystoscopy; however, she underwent an MRI pelvis for better characterization of the lesion.

**Figure 2:**
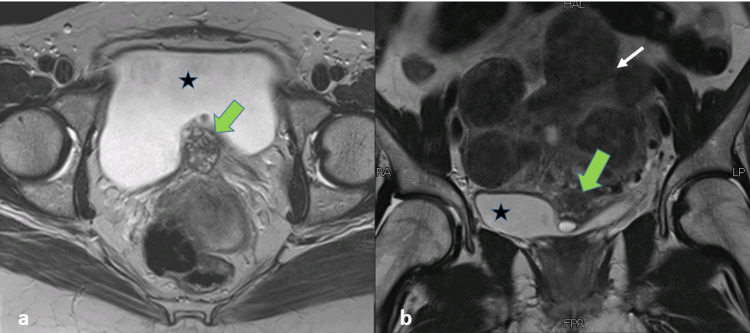
MRI pelvis - image 1 (a) Axial T2-weighted image and (b) coronal T2-weighted image demonstrate heterogeneous ill-defined predominantly T2 hypointense lesion (green arrow) along the base of the urinary bladder (star sign) with contour abnormality. Fibroid uterus are shown on the coronal view (white arrow) MRI: magnetic resonance imaging

**Figure 3:**
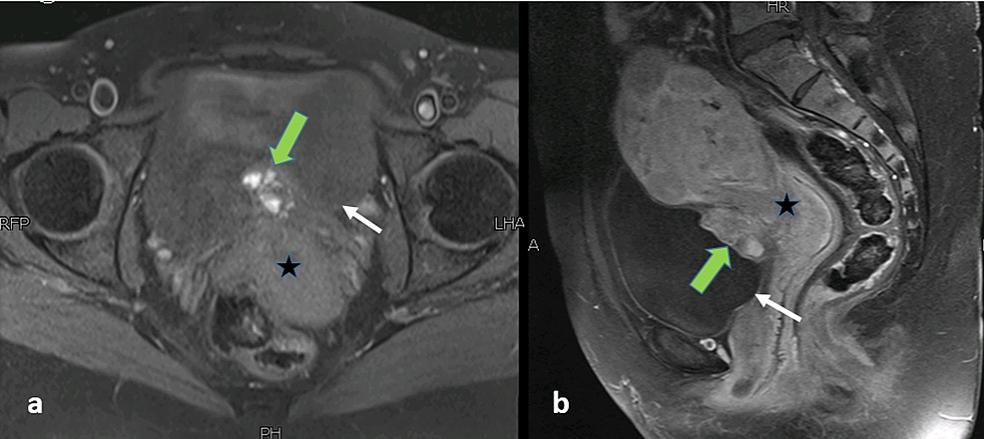
MRI pelvis - image 2 (a) Axial T1-weighted fat-saturated image showing ill-defined heterogeneously hyperintense lesion (green arrow) along the base of the urinary bladder (white arrow). (b) Sagittal post-contrast T1-weighted fat-saturated image showing patchy enhancement within the lesion (green arrow). It is closely abutting the lower uterus (star sign) MRI: magnetic resonance imaging

**Figure 4:**
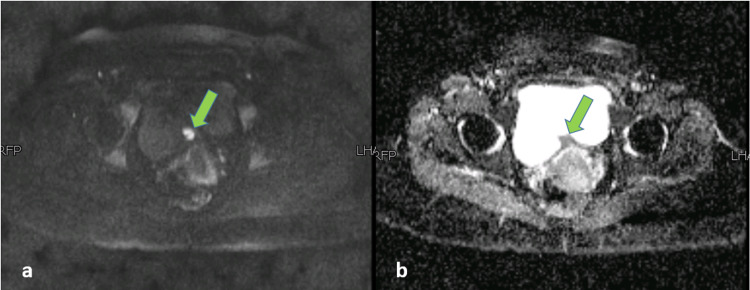
MRI pelvis - image 3 (a) Diffusion-weighted sequence and (b) apparent diffusion coefficient demonstrating the focal area of diffusion restriction within the lesion (green arrowhead) MRI: magnetic resonance imaging

The MRI pelvis showed an enlarged uterus with multiple uterine fibroids and an irregular mixed signal intensity mass along the posterior wall of the urinary bladder. This lesion was showing intravesical extension and was posteriorly in contact with the uterus with indistinct fat planes (Figure 2). On careful inspection, the lesion was found to be causing an extrinsic impression with possible muscle invasion. It measured 36 x 29 x 21 mm in craniocaudal, AP, and transverse dimensions, and the appearances were suggestive of a urinary bladder growth. However, signal characteristics of this lesion were typical of a hemorrhagic lesion, returning heterogeneously hypointense signals on T2-weighted sequence (Figure 2), heterogeneously hyperintense signals on T1-weighted sequence with patchy post-contrast enhancement (Figure 3), and focal diffusion restriction (Figure 4). Given the patient's history, MR findings were consistent with bladder endometriosis. She subsequently underwent laparoscopic resection of the bladder lesion, with the pathologic specimen showing endometriosis. Laparoscopic findings revealed overt endometriosis at the vesicouterine interface with a relatively immobile anterior wall of the uterus. Myomectomies were also performed for multiple fibroids, which were confirmed on histopathology. The patient had a good postoperative recovery and was found to be symptom-free in her routine follow-up after six weeks.

## Discussion

Endometriosis is a benign condition characterized by the presence of heterotopic endometrial tissue that structurally and functionally behaves as endometrial glands or stroma [2]. It has an incidence rate of 6% among Pakistani women as reported in the literature [2]. Urinary tract endometriosis is seen in approximately 1% of women with endometriosis, and bladder involvement is the most frequent type, occurring in 70-85% of cases [3]. It manifests in three different ways: (a) peritoneal-superficial disease, (b) endometriomas-ovarian disease, and (c) deep infiltrating endometriosis, which is the most complicated and surgically difficult form [3]. Endometriosis can occur in combination with adenomyosis (a condition in which endometrial tissue is restricted to the uterine myometrium) [4]. Endometriosis can vary significantly from large cysts (endometriomas) and solid deposits to microscopic implants [5]. The decreasing order of involvement by endometriotic deposits is as follows: ovaries, pelvic peritoneum, C-section scars (scar endometriosis), deep tissues (subperitoneal), gastrointestinal tract, bladder, chest, and subcutaneous tissues [5]. It mostly affects young females of reproductive age and may arise secondary to fluctuating levels of progesterone and estrogen [6]. In this report, we discussed a rare case of urinary bladder endometriosis.

The patients commonly present with infertility or pain-related symptoms, including cyclic pelvic pain, dysmenorrhea, dyspareunia, dysuria, and dyschezia [7]. Bladder endometriosis is most commonly associated with dysuria, frequency, haematuria, and, less frequently, urgency and bladder pain. These symptoms may get aggravated during the menstrual cycle or may have a noncyclical presentation [3]. In our case, the patient did not have urinary symptoms, which hindered the proper diagnosis of endometriosis on imaging.

Various theories have been proposed in the literature regarding the cause of endometriosis, and the most preferred one is retrograde menstruation. It has been found that during menstruation, there is spillage of endometrial cells in the anterior cul-de-sac, which is the most dependent portion, just like the posterior cul-de-sac, hence representing a favorable site for deep endometriosis. It is proposed to perform imaging before or during menstruation as the lesions become more perceptible [8].

Laparoscopic evaluation and histological confirmation are the gold standard methods for the diagnosis of endometriosis [5]. Valuable noninvasive imaging tools such as ultrasound and MRI can avoid delays in diagnosis and treatment. The first-line imaging modality to rule out any abnormality is ultrasonography, followed by MRI, which is more robust in revealing deep endometriosis [8].

On ultrasound, endometriotic nodules appear as well-defined, solid, hypoechoic, irregular masses. They may contain low-level echoes or small cystic spaces and often show no blood flow on color Doppler. Anteverted-retroflexed uterus ("question mark sign") is often seen with severe posterior compartment deep infiltrating endometriosis. The "kissing ovaries" sign signifies ovaries that are adherent to one another posterior to the uterus and are frequently seen with bilateral endometriomas [9]. Deep infiltrating endometriosis of the bladder most commonly involves the bladder base and bladder dome; the appearance of nodules can vary, including hypoechoic linear or spherical lesions, with or without regular contours involving the muscularis (most common) or submucosa of the bladder [10].

MRI is a robust imaging technique for the assessment of bladder endometriosis. Whenever there is a high suspicion of cancer, MRI is a reliable modality with its superior tissue characterization, better multiplanar capability, higher contrast resolution, and better delineation of bladder wall layers, in comparison with ultrasonography. The typical appearance of bladder endometriosis on MRI is usually of low signal intensity on T2 weighting with intermediate signal intensity on T1 weighting, and foci of high signal intensity on T1 and T2 weighting [11]. According to a study by Medeiros et al., pelvic MRI has a pooled sensitivity of 0.64 (95% CI: 0.48-0.77) and a pooled specificity of 0.98 (95% CI: 0.96-0.99) for the detection of bladder endometriosis [11]. No edge has been reported for 3.0-T MRI or gadolinium-enhanced MRI over conventional MRI [3]. In contrast, the signal characteristics of bladder malignancy on MRI are isointense compared to muscle on T1-weighted imaging, slightly hyperintense compared to muscle on T2-weighted imaging, and shows enhancement on post-contrast T1-weighted imaging [12].

Few "pearls of wisdom"/recommendations regarding the use of MRI for the diagnosis and characterization of pelvic endometriosis are presented here. (a) T1-weighted fat-suppressed sequences should be included in all-female pelvis MR examinations because this sequence is very helpful in detecting small endometriomas and differentiating them from mature cystic teratomas. Peritoneal endometriotic deposits usually appear bright on T1-weighted images, representing blood products, and hence the best sequence to pick this abnormality is the T1-weighted fat-saturated sequence. (b) It must be taken into account that endometriomas and endometriotic foci can demonstrate restricted diffusion and mimic pelvic malignancies. (c) The ancillary finding of hematosalpinx and endometriomas with "T2 shading" sign (demonstrating typical layering effect with low T2 signal) are suggestive of pelvic endometriosis. These ancillary findings were not present in our case to support the diagnosis. (d) Solid deep infiltrating endometriosis, as seen in our case, can involve the uterus, cul-de-sac, pelvic ligaments, anterior rectosigmoid colon, bladder, as well as surgical scars. The best sequence to detect deep infiltrating endometriosis and associated fibrosis is the T2-weighted MR sequence without fat suppression. These lesions demonstrate ill-defined margins and T2 hypointense signals as a result of fibrosis along with foci of T2 hyperintensity representing ectopic endometrial glands. These typical MR signal characteristics can help in establishing the diagnosis [1,13,14].

## Conclusions

This case report highlights the importance of a collaborative effort involving clinicians and radiologists in patient management. In a nutshell, our report illustrated typical MRI features of bladder endometriosis in a patient with atypical clinical presentation of the disease. Familiarity with imaging techniques and background knowledge of this rare entity are of paramount importance to reach an early diagnosis and avoid unnecessary interventions, thereby reducing morbidity and mortality among patients.
